# An Algorithm for Computing Side Chain Conformational Variations of a Protein Tunnel/Channel

**DOI:** 10.3390/molecules23102459

**Published:** 2018-09-26

**Authors:** Udeok Seo, Ku-Jin Kim, Beom Sik Kang

**Affiliations:** 1 3D Convergence Technology Center, 70 Dongnaero, Donggu, Daegu 41061, Korea; power88a@naver.com; 2 School of Computer Science & Engineering, College of IT Engineering, Kyungpook National University, 80 Daehakro, Bukgu, Daegu 41566, Korea; 3 School of Life Sciences and Biotechnology, Kyungpook National University, 80 Daehakro, Bukgu, Daegu 41566, Korea; bskang2@knu.ac.kr

**Keywords:** protein molecule, side chain rotamer, conformation, tunnel, channel, flexibility, visualization

## Abstract

In this paper, a novel method to compute side chain conformational variations for a protein molecule tunnel (or channel) is proposed. From the conformational variations, we compute the flexibly deformed shapes of the initial tunnel, and present a way to compute the maximum size of the ligand that can pass through the deformed tunnel. By using the two types of graphs corresponding to amino acids and their side chain rotamers, the suggested algorithm classifies amino acids and rotamers which possibly have collisions. Based on the divide and conquer technique, local side chain conformations are computed first, and then a global conformation is generated by combining them. With the exception of certain cases, experimental results show that the algorithm finds up to 327,680 valid side chain conformations from 12^8^~12^33^ conformation candidates within three seconds.

## 1. Introduction

Tunnels and channels in a protein molecule are the pathways for ligands (or substrates). The ligands are able to move from outside to inside cavity of the molecule or pass through the molecule by using the tunnels or channels. When a ligand reaches to the cavity, they can interact with residues in the protein molecule, where it causes functions of the protein. A channel is a pathway with two entrances from the exterior to the inside, while a tunnel has only one entrance. For studying protein functions, it is important to find them through which the ligand passes in a protein. The term *tunnels* can be replaced with *channels* in this paper, since we will suggest an algorithm which can be applied to both of tunnels and channels without modification. In the remaining part of this paper, we will use the term *tunnels*, rather than using the term *tunnels and channels*.

There have been many researches on finding tunnels in the protein molecule [1,2,3,4,5,6,7,8,9,10,11,12,13,14,15,16,17,18,19,20,21,22,23,24,25]. To find the tunnels, geometric information on atoms in the molecule must be known. The Protein Data Bank (PDB, http://www.rcsb.org) provides such geometric information as snapshots of a molecule at the static state. In this case, the relative positions of the atoms in the molecule are static, and the tunnel can be computed for the atoms with fixed positions.

In the natural state, protein molecules are flexible, since the side chain of each amino acid can be rotated, and the backbone is flexible. Therefore, the shape of the tunnel in the molecule can be changed according to the changed atom positions in the molecule. When the tunnel shape is changed, the ligand types that pass through it also can be changed; thus, computing the shape changes of a tunnel is important.

In particular, when the target of a chemical is inside the protein, the accessibility of the chemical to the target is essential to the function of the protein. For example, DosT, an oxygen sensor protein, from *Mycobacterium tuberculosis* has a tunnel for oxygen to a heme inside the protein, while its paralog, DosS has a narrower tunnel. Rotation of the side chain of a residue at the tunnel in DosS can change the tunnel even narrower not to pass an oxygen molecule, and as a result, DosS becomes a redox sensor unlike DosT [26]. In cases of enzyme, the shape of the tunnel restricting the size of substrate provides substrate specificity of the enzyme. Changes in the shape of the tunnel affect the activity of the enzyme because access to the target site of the substrate increases the local concentration of the substrate. Tunnel shape changes can be also applied to ligands for cell signaling, inhibitors targeting the enzyme active site, allosteric modulators, and so on. Thus, it will provide valuable information for drug discovery and determination of molecular mechanisms for enzymes and sensor proteins.

So far, most research related to the shape changes of the tunnel is either on computing them for each snapshot of protein molecule captured during sequential time period or computing them by using the molecular dynamics [9,10,11,12,13,14,15,16,17,18,19,20,21,22,23,24,25,27,28]. For previous research, the tunnel shape changes are tracked for a sequence of snapshots.

For the protein molecule with side chain flexibility, we suggest an algorithm to compute valid conformational variations for the amino acid side chain rotamers composing a tunnel. Our algorithm is summarized as follows. Given a tunnel, the amino acids composing it are extracted. For those tunnel adjacent amino acids, we remove the side chain rotamers that cause invalid conformations. Based on two types of graphs and their maximal cliques, the amino acids and rotamers are classified as two groups: amino acids and rotamers in one group are guaranteed to have no collisions each other, while those in the other group possibly have collisions. After computing the local valid conformations from each maximal clique, the global valid conformations are generated based on the divide and conquer method. As one of the applications of the suggested algorithm, we show the way to compute the maximum bottleneck of the flexibly deformed tunnel. Experimental results show the computational time efficiency of our algorithm.

Graphs have been widely used for side chain prediction (or side chain packing) problems [29,30,31,32,33,34,35,36], where the problem is known as NP-hard [37,38]. For the side chain prediction, graphs and cliques have been used to find the rotamer conformation that minimizes the energy between atoms. To solve the problem, many variations of graphs and graph search methods have been used, and most of previous research focused on solving the combinatorial optimization.

In this paper, though we suggest a method that uses graphs and maximal cliques like as the previous works on side chain prediction problems, the output of our method is different from them. Rather than finding one solution that corresponds to combinatorial optimization, our method finds every valid rotamer conformation. The purpose of our research is computing flexibly deformed tunnels from a given initial tunnel, so we need to compute every valid rotamer conformation rather than finding one optimized conformation. For the efficient finding of all valid conformations of the side chain rotamers, we suggest ways to compute the local valid conformations from the graph. We also present a way to combine those to global valid conformations.

Both of our algorithm and molecular dynamics simulation can produce a valid conformation, but there are differences in possibility or existing time. A sequence of molecular dynamics snapshots are the conformations with various energy states and it can provide the most possible conformation, which exists for the longest time in a given time period. Our algorithm focused on fast finding of every valid rotameric conformation around a tunnel, so it is useful to decide the maximum bottleneck of the varied shapes of a tunnel starting from the same position in the protein. Although the shapes of a tunnel simulated by our method with a fixed backbone would be different from those generated by molecular dynamics simulation, our algorithm shows a specific application that it can be used with interactive computation time performance for the most of cases.

This paper is organized as follows: in Section 2, related works are discussed. The geometric features of the protein molecule and amino acids are presented in Section 3. In Section 4, we present an algorithm for computing every valid rotamer conformation in the tunnel adjacent amino acids. By using the valid conformations, we present a method to compute the maximum bottleneck of the tunnel, which decides the maximum size of the ligand that can pass through the tunnel, in Section 5. The experimental results are discussed in Section 6, and we conclude this paper in Section 7.

## 2. Related Works

There has been previous research on computing or tracking tunnels in the protein molecule, and there exist many software tools or algorithms [1,2,3,4,5,6,7,8,9,10,11,12,13,14,15,16,17,18,19,20,21,22,23,24,25]. Those tools or algorithms find the cavities and tunnels efficiently, and the ligand size can be decided for each tunnel. The methods for tunnel finding are applied for the protein molecule in a static state [1,2,3,4,5,6,7,8], while those for tunnel tracking are used for deformed tunnels in a single protein molecule under the time-varying situation [9,10,11,12,13,14,15,16,17,18,19,20,21,22,23,24,25].

There are good survey papers on computing tunnel finding algorithms [27,28]. Simoes et al. [28] classified the cavity into void, pocket and channels, and then classified pockets as cleft/groove, invagination, and tunnels. A number of methods are shown for tunnel finding and tracking under the time-varying situation [9,14,15,16,17,18,19,20,21,22,23], and most of them use the molecular dynamics result for tracking the changed shape of tunnels.

Proteins have a flexibility which causes shape changes along the time axis. Beneš et al. [10,11] proposed a method to track the dynamic tunnel by using a Voronoi diagram of the protein. For a sequence of snapshots of protein molecule, they compute a Voronoi diagram for each snapshot. From the initial tunnel, they choose the nearest Voronoi edges to it, and then construct the modified tunnel. Based on their method, in CAVER 3.0 [12,13], the tunnel updates for each snapshot is computed in parallel. Rather than computing the whole tunnel, it efficiently updates the tunnels by choosing the similar pathway to the initial tunnel.

Raunest and Kandt [16] presented dxTuber that is a grid-based cavity simulation method based on protein and solvent residence probabilities, where those probabilities are derived from molecular dynamics simulations. They convert solvent and protein trajectories into density voxels, and then detect internal solvent voxels (ISV) through scanning density voxels in the directions of principal axes. After they find ISVs, they group ISVs into buried cavities, surface clefts, and tunnels.

Craig et al. [15] presented PocketAnalyzer^PCA^, which uses principal component analysis and clustering for the detected pockets. For each dynamically changed protein structure derived from molecular dynamics, they detect pockets based on grid method. They encode the pocket shape as a row vector of integers 1 and 0, by representing the grid points as included in the pocket or not. A pocket shape matrix is composed by merging those row vectors, and derives the principal components from the matrix. Then, the dominant deformation modes of dynamic pocket shape are computed by projecting the row on those principal components.

Ashford and et al. [17] showed Provar that provides a way to analyze and visualize the pockets in a flexibly deformed protein structure. Rather than presenting a novel way to compute the pockets from the deformed protein structure, they focused on the post-processing of the outputs from existing various pocket finding software tools. They discussed the difficulties of comparing predicted pockets from a single protein structure when those pockets are detected by using different software tools. They also showed the difficulties of comparing the pockets detected from conformational variants from a protein structure. To solve the difficulties, they find the pocket-lining atoms and side chains by the probability scores based on the cut-off distance of each atom from every pocket derived from different software tools. Then, the atoms within a specified probability range are colored as pockets.

Lindow et al. [19,20] developed a tool to compute dynamics of cavities based on the computation of Voronoi diagrams of spheres. They derived geometric paths from static protein structures at each time step based on Voronoi diagrams of spheres, where each static structure is decided by molecular dynamics. Then, they associated each path with a cavity, and traced the paths over time by computing the intersection volume of two cavities.

Paramo et al. [21] presented trj_cavity for detecting cavities. They represent the 3D grid system with voxels. They map the atoms to voxels, and then incrementally detect the cavities from one of voxels inside a cavity. By checking the neighbors of the voxel in a cavity, they find a connected cavity. When there is no more empty voxels in the cavity, they start to detect new cavity. They detect cavities for each snapshot of molecular dynamics simulation, and consider a cavity is identical if there are overlaps between two cavities in the sequence of snapshots.

Desdouits et al. [22] propose an algorithm for tracking the dynamics of cavity topology changes along the sequential frame of molecular dynamics. They applied Principal Component Analysis (PCA) to analyze the characteristics of the evolution of cavity geometry, and described the cavity evolution by using the PCA components.

Laurent et al. [23] presented Epock, where it focuses on tracking the identical cavity along the sequence of frames from molecular dynamics simulation. Rather than detecting cavities, they use the cavities computed in a priori by using other software.

Vonasek et al. [24,25] presented sampling-based motion planning method to detect tunnels in a protein molecule. By using the concept from robot motion planning and Voronoi diagram, they used the Rapidly Exploring Random Tree algorithm to extract the empty space in each molecular dynamics frame of the protein. They represented the empty space in the protein molecule as a tree structure, and updated it according to the disappearance, split, or merge of the empty space in the next frame.

## 3. Preliminaries

By using the atom center positions provided by the PDB file and van der Waals radius [39], we can represent each atom as a sphere with a specific center position and radius in a 3-dimensional space. It is general to represent atoms to spheres for the geometric approaches in analyzing protein structure. When two atoms have a covalent bond, they are represented as two intersecting spheres, while the corresponding spheres of non-bonded atoms do not intersect.

The atoms in an amino acid are classified as two parts: main chain and side chain. In every amino acid, the main chain part is composed of the same four atoms (N, Cα, C, O), where each neighbor atoms are linked by covalent bonds. The main chains of amino acids sequentially compose a polypeptide chain.

In the side chain, the atoms are hierarchically structured with each hierarchy sharing the same rotation axis and angle, and this produces the side chain flexibility. The atom Cα in the main chain has covalent bond with the atom Cβ in the side chain. The line passes through the centers of Cα and Cβ works as the first rotation axis, by which the whole side chain is able to rotate around this axis, where in the lower hierarchy similarly rotation occurs. According to the amino acid type, there can be 0 to 4 rotation axes (Cα-Cβ, Cβ-Cγ, Cγ-Cδ, and Cδ-Cε) in a side chain. The rotation angle for each rotation axis is given to satisfy energetically favored arrangement of the adjacent atoms attached to the axis. All atomic positions of the side chain adopting the rotation angles determine the specific conformation of the side chain. There are numerous possible conformations for a given side chain. A few conformations occur more frequently than others, and are called rotamers.

Except the case when two atoms have a covalent bond, the intersection between corresponding spheres is not allowed. We define the case when two atoms without a covalent bond have intersection as a collision. For two non-bonded atoms A_1_ and A_2_ with the center point (x_i_, y_i_, z_i_), i = 1, 2, and radii r_i_, the collision occurs when the following is satisfied:

(x_1_ − x_2_)^2^ + (y_1_ − y_2_)^2^ + (z_1_ − z_2_)^2^ < (r_1_ + r_2_)^2^.

We consider it as a valid conformation only when there is no collision between two rotamers of different amino acids. Bounding Sphere Volume (BSV) [40] is a spherical area that containing an amino acid with its every rotamer from all hierarchies. For the side chain atoms at the lowest hierarchy, we construct a bounding sphere first. In the bottom up direction, for each hierarchy, we compute the bounding sphere which contains the rotation of the bounding sphere in the lower level. In this way, at the highest hierarchy, we have a bounding sphere which contains every rotamer in lower hierarchies. When we represent each amino acid with two non-intersecting BSVs, it is guaranteed that there is no collision between two amino acids for any rotamer conformation. We can reduce the computation time by checking collisions between BSVs rather than rotamers. If two BSVs B_1_ and B_2_ do not intersect, the rotamers in B_1_ and those in B_2_ do not have collisions. On the other hand, using BSVs results less accuracy. Though B_1_ and B_2_ have an intersection, there are possibilities that two rotamers from B_1_ and B_2_ have a valid conformation without collision. In this reason, when B_1_ and B_2_ intersect, we check every pair of rotamers from B_1_ and B_2_, to finally decide if there are valid conformations. BSVs also are used to decide the tunnel adjacent amino acids. For the amino acids whose BSVs do not intersect with the tunnel, we can exclude them from the tunnel adjacent ones.

**Figure 1:**
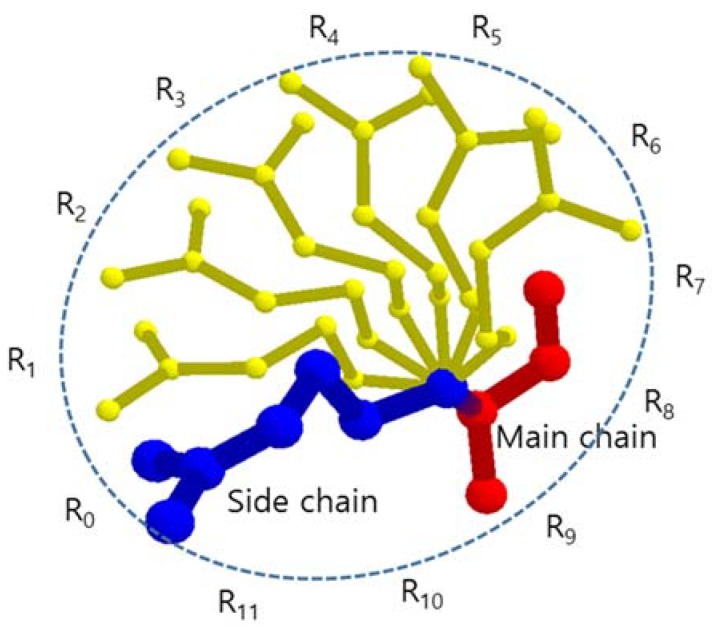
The side chain and its rotamers that are represented by atom center points, where R_0_ is identical to given side chain.

Since the atoms bonded to a carbon are at the tetrahedral position, the side chain prefers the staggered conformation of neighbor atoms to avoid steric hindrance. Thus, the favorable rotamers are positioned every 120° around the rotation axis, and there are three rotamers for each rotation axis [41,42]. A long side chain with four steps of rotation axes has 3^4^ (81) possible conformations. However, the rotation axis, which is most effective to determine a BSV of an amino acid, is the axis of Cα-Cβ. In this paper, we assumed that each side chain has one rotation axis Cα-Cβ to reduce the amount of calculation and the conformations of a side chain are generated by rotating the whole fixed side chain every 30° around the rotation axis Cα-Cβ considering possible spatial positions of side chain atoms generated by other rotation axes (Figure 1). Then, each amino acid has 12 conformations and when we consider n amino acids in a tunnel, there are up to 12^n^ rotamer conformation candidates. Since the volume of hydrogen atoms attached to C, N, O, and S atoms is relatively small and ignorable compared to the BSV, we did not consider the hydrogen atoms.

## 4. Suggested Algorithm

The suggested algorithm computes every valid conformation of side chains in tunnel adjacent amino acids, which can be found from only one snapshot of the protein molecule. The algorithm overview is presented in Algorithm 1. We can obtain atom positions, covalent bond relation, rotation axis, amino acid information, etc. from a PDB file. They are input to the algorithm with tunnel information.

Amino acids which possibly affect the given tunnel are selected and all of their rotamers are added to the set S. Then, we remove the side chain rotamers which collide with main chains and other static atoms. For the amino acids with remaining rotamers, the collision graph and the collision free graph are constructed. By finding maximal cliques from those graphs, we can group the amino acids which do not collide at all, and generate the valid rotamer conformations for the amino acids possibly have collisions each other. For each subset of amino acids, rotamer valid conformations are found. Then, the valid rotamer conformations for all tunnel adjacent amino acids are computed by combining them.


**Algorithm 1**

Input: Protein Molecule M, a tunnel HStep 1: S ← {amino acids that are adjacent to H, and their rotamers}Step 2: S ← S–{invalid rotamers of the amino acids in S}Step 3: CG ← collision graph for amino acids in S
Extract maximal cliques from CG
//if two amino acids are in different maximal cliques in CG, 
//they do not have collisions in any caseStep 4: For each maximal clique of CG,
FG_k_ ← collision free graph for rotamers in each maximal clique of CG
Extract maximal cliques from FG_k_
//if rotamers of different amino acids have an edge 
//in a maximal clique in FG_k_, then they have no collisionStep 5: Compute local valid conformations from each maximal clique of FG_k_Step 6: Combine the local valid conformations to global valid conformationsStep 7:Output global valid conformations

### 4.1. Step 1: Extraction of Amino Acids That Are Adjacent to a Tunnel

**Figure 2:**
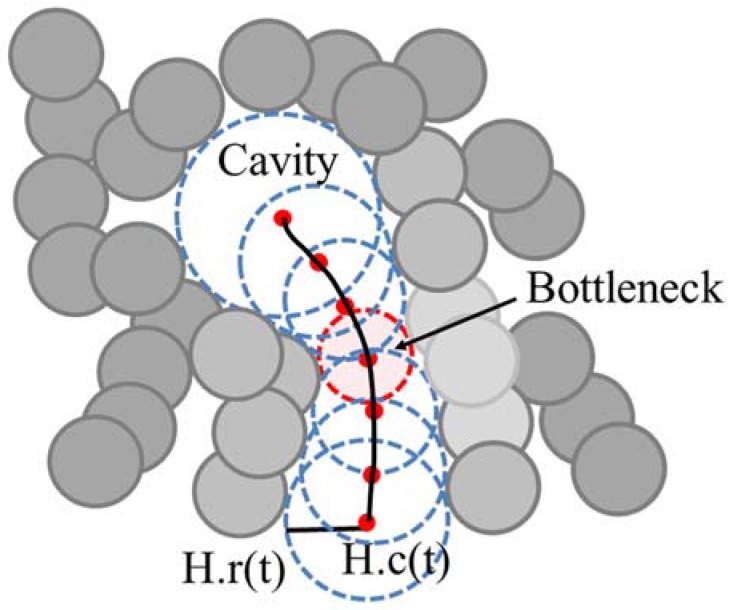
Example of the tunnel in the protein molecule and its bottleneck.

We represent the tunnel H(t), 0 ≤ t ≤ 1, as the center trajectory H.c(t) and the radius H.r(t) of the moving empty ball, which represents the tunnel as a swept volume (Figure 2). When the radius of empty ball is locally minimum, we denote it as a bottleneck of the tunnel. When there are one or more bottlenecks in a tunnel, the minimum bottleneck restricts the size of a ligand that can reach to the inside cavity.

The amino acids adjacent to tunnel H can be defined as two types:

(1) The amino acids that contact with the tunnel H in the initial state
(2) The amino acids whose side chain rotamers possibly collide with the tunnel H

The original definition of the tunnel implies that the tunnel has no collision with atoms, and only some atoms contact the tunnel. However, for an amino acid which was apart from the tunnel in the initial state, its side chain rotamer possibly collides with and affects the tunnel. So, we consider both types of amino acids as tunnel- adjacent ones.

For each amino acid A_i_, 1 ≤ i ≤ n, we compute BSV(A_i_) that contains every side chain rotamer of A_i_. Then, the set of tunnel adjacent amino acids, S, is computed as follows:

S = {A_i_ with its rotamers, which satisfies BSV(A_i_) ∩ H(t) ≠ ∅, 1 ≤ i ≤ n}. (1)

**Figure 3:**
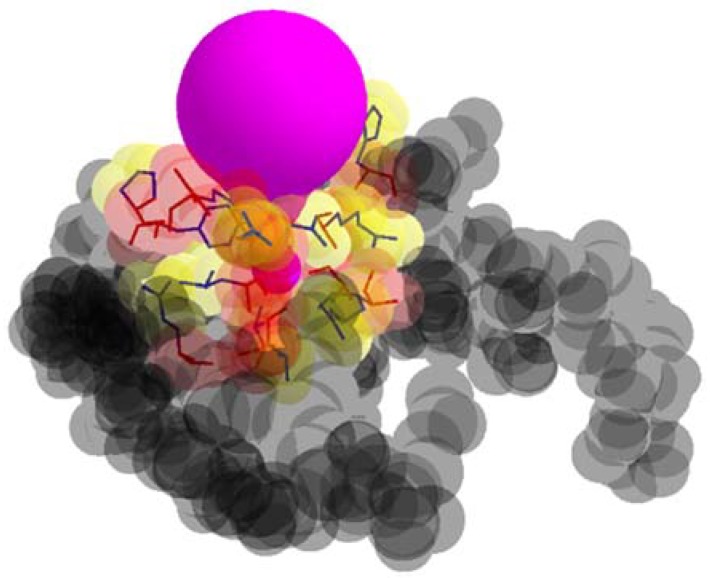
A tunnel (color purple), tunnel adjacent amino acids, and remaining amino acids (color gray).

In Figure 3, the examples of a tunnel and tunnel adjacent amino acids are presented.

### 4.2. Step 2: Removing Invalid Rotamers

We assumed that there are 12 rotamers for each tunnel adjacent amino acid in S, and that the remaining amino acids are static. For each tunnel adjacent amino acid, one of the rotamers corresponds to the initially given side chain in the PDB file.

In the following cases, rotamers are considered to be invalid:

- Case 1: a rotamer that collides with main chains
- Case 2: a rotamer that collides with the amino acids not in S

**Figure 4:**
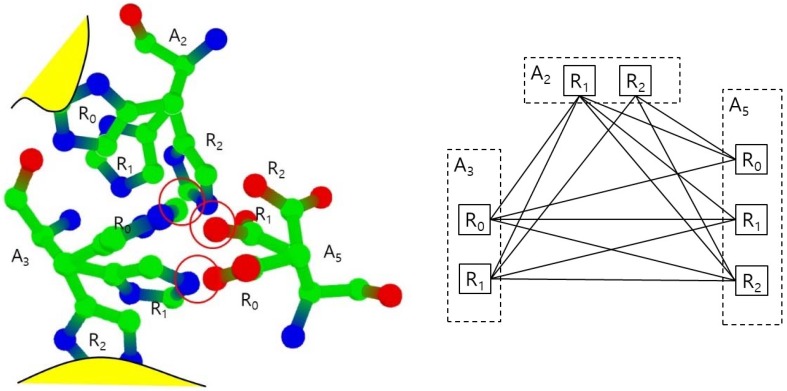
Collisions between rotamers (**left**) and the corresponding collision free graph (**right**).

In the normal status, both cases do not happen, so we consider the rotamers in those cases as invalid. We also remove those invalid rotamers from the set S. In Figure 4, the rotamers A_2_.R_0_ and A_3_.R_2_ are invalid since they collide to main chain or amino acids which are not in S.

### 4.3. Step 3: Computing Amino-Collision-Cliques

A CG graph is used to group the amino acids whose rotamers possibly collide with each other. Before computing CG, we first compute a temporarilly used graph TG for the computation time efficiency, and then revise it to CG. The temporary collision graph TG(V,E) is constructed as follows, where V and E denote a vertex set and an edge set, respectively:

V = {A_i_}, A_i_ ∈ S, where 1 ≤ i ≤ n (2)

E = {(X, Y) | X, Y ∈ V and BSV(X) ∩ BSV(Y) ≠ ∅}(3)

For each amino acid pair (X,Y), which shares an edge in TG, we check if a rotamer from X and another one from Y have a collision. For an amino acid A, we represent its rotamers as A.j. If any pair of rotamers has a collision, then an edge between X and Y is added to the edge set of CG. The collision graph CG(V,E) is as follows:

V ={A_i_}, A_i_ ∈ S, where 1 ≤ i ≤ n (4)

E = {(X, Y) | X, Y ∈ V and there exist one or more X.a ∩ Y.b ≠ ∅}(5)

After CG is constructed, we compute the set of maximal cliques by using Bron-Kerbosch algorithm [43]. The clique is a complete subgraph, and the maximal clique is a complete subgraph which is not included in another complete subgraph.

We denote the maximal cliques of CG as amino-collision cliques. If two amino acids are in the same amino-collision-clique, they always have one or more rotamer pairs which collide each other. If two amino acids A_i_ and A_j_ are in different amino-collision-cliques C_1_ and C_2_ and A_i_ and A_j_ are not in C_1_ ∩ C_2_, then they are guaranteed to have no collisions for any pair of rotamers.

### 4.4. Step 4: Computing a Collision Free Graph for Each Amino-Collision-Clique

For each maximal clique in CG, we construct a collision free graph, where it is used to find the valid rotamer conformation for the amino acids that possibly have collisions. For every pair of amino acids in amino-collision-clique, every rotamer pair is checked for collisions.

Let us denote the amino-collision-cliques of CG as C_1_, C_2_, …, C_m_. Then, for each clique C_k_, 1 ≤ k ≤ m, a collision free graph FG_k_ (V_k_, E_k_) is constructed by Algorithm 2.


**Algorithm 2**
For k = 1 to m do begin For each amino acid A_i_ in C_k_,  Add every rotamer A_i_.j to V_k_ For each rotamer pair A_x_.a and A_y_.b, x ≠ y and A_x_, A_y_ ∈ V_k_ do  If A_x_.a ∩ A_y_.b = ∅ then   Add (A_x_.a, A_y_.b) to E_k_
end

### 4.5. Step 5: Computing Local Valid Rotamer Conformations

The collision free graph FG_k_(V_k_, E_k_) for amino-collision-cliques C_k_ represents the valid rotamer conformation for the amino acids in C_k_. If we can find a maximal clique from FG_k_ whose number of vertices is identical to the number of amino acids in FG_k_, then it corresponds to a valid rotamer conformation for the amino acids in C_k_. There is no valid conformation for those in C_k_, otherwise.

Figure 4 shows the example of the collision free graph for the amino-collision-clique with vertices for amino acids A_2_, A_3_, and A_5_ and their valid rotamers. Each amino acid is assumed to have three rotamers: R_0_, R_1_, and R_2_, where A_2_.R_0_ and A_3_.R_2_ are invalid rotamers. The rotamer pairs (A_2_.R_2_, A_3_.R_0_), (A_2_.R_2_, A_5_.R_1_,), and (A_3_.R_1_, A_5_.R_0_) have collisions, respectively. Other pairs have no collisions, so the collision free graph is constructed with an edge set excluding only (A_2_.R_2_, A_3_.R_0_), (A_2_.R_2_, A_5_.R_1_), and (A_3_.R_1_, A_5_.R_0_). In this collision free graph, we find all maximal cliques. For example, there are three maximal cliques which includes the edge (A_2_.R_1_, A_3_.R_0_) such as (A_2_.R_1_, A_3_.R_0_, A_5_.R_0_), (A_2_.R_1_, A_3_.R_0_, A_5_.R_1_) and (A_2_.R_1_, A_3_.R_0_, A_5_.R_2_). There are six maximal cliques with three vertices. There also is only one maximal clique with two vertices: (A_2_.R_2_, A_5_.R_0_). In this case, there is no valid conformation including A_2_.R_2_ and A_5_.R_0_, since any rotamer from A_3_ will have collisions with A_2_.R_2_ or A_5_.R_0_.

### 4.6. Step 6: Combining the Local Valid Rotamer Conformations to Global Ones

For finding the valid rotamer conformations for all the amino acids in S, we have to combine the valid conformations generated from the cliques in FG_k_. Let us denote the set of maximal cliques found in the collision free graph FG_k_ as a set Q_k_. Then, the valid conformations for each Q_k_ can be computed.

**Figure 5:**
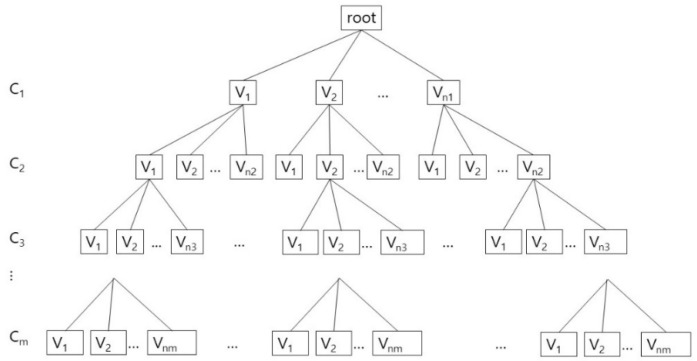
The combining process of the valid conformations V_j_, 0 < j ≤ n_j_, for the rotamer clique C_i_, 0 < i ≤ m.

After generating an array whose size is the number of tunnel adjacent amino acids, we put the rotamer identification numbers of those maximal cliques in the array. According to the intersection between two different maximal cliques, we can decide the rotamers which must be shared by two different collision free graphs. The final valid conformation is computed with backtracking. In Figure 5, we show the tree structure for the valid conformations in each clique. For each clique C_i_, 0 < i ≤ m, let us assume that there are n_i_ valid conformations: {V_1_, V_2_, …, V_ni_}, which are generated from Q_k_. Each valid conformation consists of an one-dimensional vector, whose size corresponds to the number of adjacent amino acids: (x_1_, x_2_, …, x_n_), where x_i_ is either a rotamer identification number in the valid conformation or −1 for the case when the corresponding amino acid is not included in the clique. For example, if there are 6 tunnel adjacent amino acids, and the clique contains three amino acids among them, and has a valid conformation: A_2_.R_1_, A_3_.R_0_, A_5_.R_0_, then the valid conformation V is (−1, 1, 0, −1, 0, −1).

A tree structure with searching and pruning for combining process is well-known and widely used, but for clearly showing its usage for our algorithm, we explain about it with implementation details. The combining process can be considered as a depth first search of a tree from the root with the pruning steps, if necessary (Figure 5). Whenever visiting a node N, an incrementally composed valid conformation that is the result of combination of every valid conformations of its ancestors, preVC, is given. If preVC is not compatible with the valid conformation of N, currentVC, then we do not visit the subtree rooted N. If preVC is compatible to currentVC, then we combine preVC and currentVC to compose a new incremental valid conformation, updatedVC. The new conformation updatedVC is given to N’s children when they are visited. If there are no children of N, then output updatedVC as a global valid conformation.

Though the combining process can be considered as depth first search of a tree, but actually we do not have to use the tree structure in the implementation. If we represent the cliques and their valid conformations as nodes in a tree, the number of nodes is represented as the product: n_1_n_2_…n_m_. In this reason, rather than physically constructing a tree in Figure 5, we implement the combining process by using the recursive calls of the function. By this way, we use the memory space that increases linearly to the number of valid conformations in cliques: C_1_.V_1_, C_1_.V_2_, …, C_1_.V_n1_, C_2_.V_1_, C_2_.V_2_, …, C_2_.V_n2_, …, C_m_.V_1_, C_m_.V_2_, …, C_m_.V_nm_.

The algorithm to combine the local valid conformations to a global valid conformation is presented as Algorithm 3. In the main function, the function Combine is called as Combine (1, j, preVC), where 0 < j ≤ n_1_ and preVC is initialized as the integer array (−1,−1, …, −1).


**Algorithm 3**
Combine (int cur_level, int cur_VC, int preVC[ ])//cur_level and cur_VC: pointing the VC which are visited now//preVC: the combined valid conformations for the ancestorsBegin  currentVC = C_cur_level_.V_cur_VC_;  //combine the preVC and currentVC to updateVC  //AdjAminoNum is the number of tunnel adjacent amino acids  for i = 0 to AdjAminoNum do     if (currentVC[i] > 0 and preVC[i] > 0 and currentVC[i] ≠ preVC[i])      return; //currentVC and preVC is not compatible    else if (currentVC [i] == preVC[i] or currentVC[i] == −1)       updatedVC[i] = preVC[i];    else (preVC[i] == −1)      updatedVC[i] = currentVC [i];  if (cur_level == m) output updatedVC and return;   next_level = cur_level + 1;  for j = 1 to n_next_level_ do    Combine(next_level, j, updatedVC);End

## 5. The Maximum Bottleneck of Deformed Tunnels

From the side chain conformations produced from the algorithm in Section 4, we can compute the deformed tunnels. The tunnel finding algorithm [8] is repeatedly applied to compute each tunnel. Among those minimum bottlenecks of tunnels, the maximum bottleneck is found and it decides the maximum size of the ligand when the tunnel shape is flexibly deformed.

A tunnel H(t) is represented as a set of empty balls with a center trajectory H.c(t) and radius H.r(t) (Figure 2). A bottleneck in the tunnel, H(t*), is defined as follows:

H(t*), where H.r(t* − ε) > H.r(t*) and H.r(t* + ε) > H.r(t*).(6)

For each valid side chain conformation, we can compute the tunnel H’(t) which is the variation of the tunnel H(t). After we compute the minimum bottleneck radius from each tunnel H’(t), we choose the maximum value from them. Then, the maximum value is the maximum size of the ligand that can pass through the flexible tunnels which is produced from H(t) (Refer to Algorithm 4).


**Algorithm 4**
Input: Flexible tunnels H’(t) produced from tunnel H(t)Step 1: For each tunnel H’(t),
1.1 Generate sphere set according to the conformation 
1.2 Apply tunnel finding algorithm [8] and compute the tunnel H’
1.3 For the tunnel H’, find the minimum bottleneck and add it to BStep 2: Output the maximum value from B.

## 6. Experimental Results

The suggested algorithm was implemented using Microsoft Visual C++ and OpenGL. The experiments were performed at a PC with a CPU Intel Core i5-6200U 2.6 GHz, and 8 GB RAM. For the tests, we used 22 tunnels inside nine protein molecules which were computed by the method proposed by Kim et al. [8]. For computing tunnel geometry and valid conformation, we used two programs which are separately executed. The tunnel computing program of Kim et al. was executed at a PC with a CPU Intel i5-4570 3.2GHz, 8GB RAM, and NVIDIA GeForce GTX760. The protein molecules were downloaded from the PDB website (http://www.rcsb.org).

In Table 1, we present the number of valid rotamer conformations for the specific tunnel in a protein molecule and the computation time in seconds. For the most of tunnels, we found up to 76,800 valid conformations in 1.06 s. For the cases of tunnel Id. 215 and 298 of PDB Id. 2OAR, around 300,000 valid conformations were found and it took less than 3 s for the computation. The case of tunnel Id. 334 of PDB Id. 1DDZ shows the highest diversity. More than 3,000,000 valid conformations were found, and the computation time was around 40 s.

Most of the cases, the experimental results show the efficiency of the computation time. The number of valid rotamer conformations is somewhat related to the computation time. However, there is no relationship between the computation time and the number of conformations in rotamer-cliques. Unexpectedly, the number of valid conformations is not much related to the number of tunnel adjacent amino acids, either. Regardless of the number of tunnel adjacent amino acids, the number of valid conformations is decided. For the case of tunnel Id. 72 of PDB Id. 1A52, the number of tunnel adjacent amino acids is 33 and the number of valid conformations is 17,280. Comparing to it, tunnel Id. 215 of PDB Id. 2OAR has 19 tunnel adjacent amino acids, but the number of valid conformations is 327,680. It seems that the size and shape of the side chains in the tunnel adjacent amino acids are more important for calculating valid conformations than its number of amino acids.

**Figure 6:**
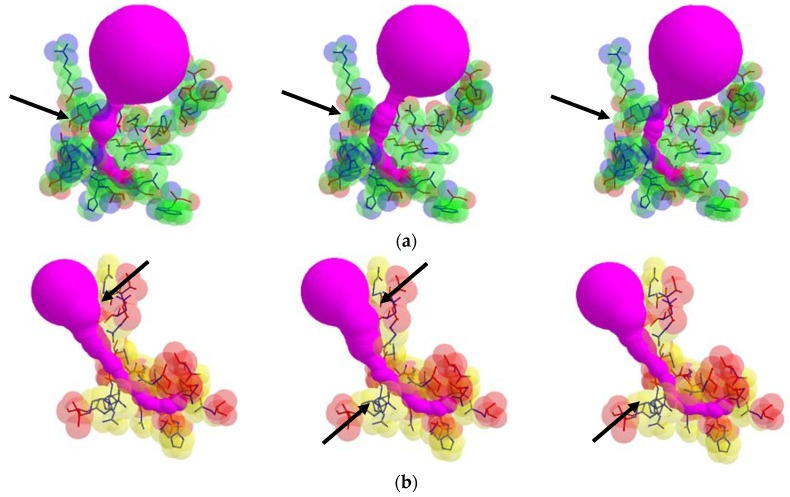
Tunnel shape changes with respect to different valid rotamer conformations, where arrows indicate changes of the tunnel shape: (**a**) tunnel Id. 138 of PDB Id. 1EA1 and (**b**) tunnel Id. 140 of PDB Id. 1DDZ.

From each valid rotamer conformation, we computed the deformed tunnel by applying the tunnel computing algorithm. The initial input tunnel shape is deformed according to the valid conformation. From every deformed tunnel shape, we obtained the bottlenecks and found the maximum bottleneck among them. By applying the suggested algorithm, we computed the valid rotamer conformations, and show two examples for the corresponding tunnel shape changes in Figure 6.

**Figure 7:**
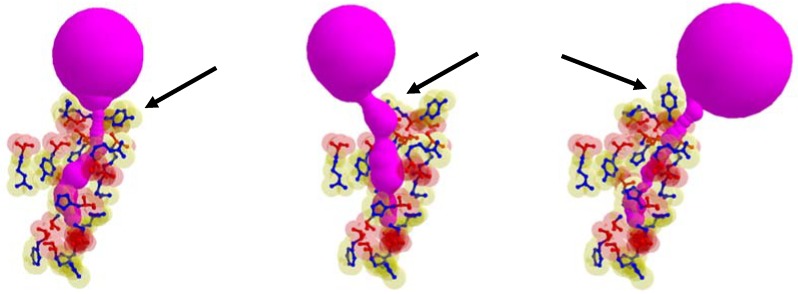
The case when the position of tunnel entrance is changed due to the different rotamer conformations, where arrows indicate changes of the rotamer (tunnel Id. 76 of PDB Id. 1MQF).

In some cases, the position of the entrance to the identical cavity was varied due to the path of tunnel is varied according to the rotation of the side chain. In Figure 7, the case when the tunnel entrance is changed due to the different rotamer conformations for the tunnel Id. 76 of PDB Id. 1MQF. For the tunnel Id. 148 of PDB id. 1B44 and tunnel Id. 51 of PDB Id. 3RLR, the rotamers which do not contribute to the tunnel shape changes are in the tunnel adjacent amino acids. We cannot extract the tunnel amino acids exactly, since it is hard to know how tunnel shape changes and which side chain rotamer possibly contributes to the deformed tunnel in advance.

In Table 2, we present the size of the maximum bottleneck of the deformed tunnels and its computation time. For each valid conformation, we applied the tunnel detection algorithm [8], and derived the deformed tunnel information. From those tunnels, we computed the maximum bottleneck. In Table 2, only the column ‘Tunnel computation time’ was measured through the tunnel detection algorithm. Usually different tunnel detection algorithms produce different tunnels though the tunnel starts from the identical position (cavity) in the protein [17,27]. The tunnels we used can be different from those derived by other software. The suggested valid conformation detection algorithm can be applied to tunnels generated from any software, if the software provides the geometric information on starting cavity and the tunnel.

The cases of tunnels Id. 215 and Id. 298 of PDB 2OAR and tunnel Id. 334 of PDB Id. 1DDZ, of which the numbers of valid conformations are greater than 100,000, are excluded from the maximum bottleneck computing since the tunnel computation takes about 1 s per a conformation. For other cases, we present the computation time for tunnels and finding the maximum bottleneck in seconds. For each tunnel, we compute all valid conformations first. Then, we construct the deformed tunnel from each valid conformation, and find the bottleneck which is the smallest empty ball of it. After deciding the bottleneck for each deformed tunnel, we find the maximum bottleneck from those. The computation time for the deformed tunnels and their maximum bottleneck is almost linear to the number of valid rotamer conformations.

**Figure 8:**
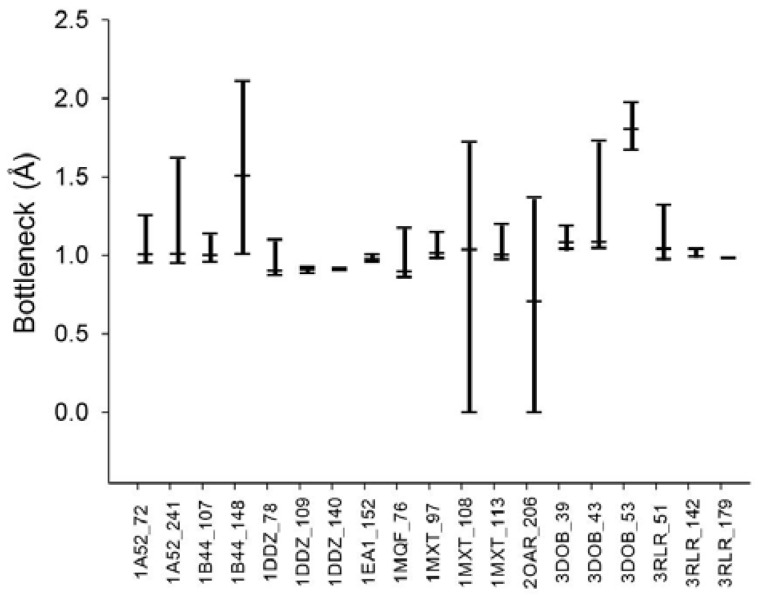
Dynamics of the tunnel bottleneck with valid rotamer conformations. The top and bottom bars indicate the maximum and minimum bottleneck sizes, respectively. The middle bar shows the average bottleneck size in the deformed tunnels.

From the various tunnel shapes, we can obtain the bottleneck in each shape and the range of bottleneck changes by the minimum and maximum bottleneck values. In Figure 8, we present the minimum, maximum and mean of bottleneck values for each tunnel. The difference between the maximum and minimum values suggests dynamics of the tunnel bottleneck by rotating side chains in the tunnel. The low bottleneck value such as 0 implies that the tunnel could be completely blocked or the cavity is disappeared. If the side chain can be rotate freely, the mean value represents the average opening of the tunnel in a given time period suggesting the accessibility to the cavity through the tunnel. For the cases of tunnel Id. 140 of PDB Id. 1DDZ and tunnel Id. 179 of PDB Id. 3RLR, the maximum and minimum bottlenecks have little difference. The tunnels in these cases do not have big difference of shapes around the bottleneck.

## 7. Conclusions

In this paper, a novel algorithm to find every valid conformation for the side chain rotamers in tunnel adjacent amino acids was presented. The algorithm selected the amino acids that possibly affect the tunnel shapes as tunnel adjacent ones. By using the graph structure, the tunnel adjacent amino acids are grouped by the collision possibility. Then based on a divide and conquer technique, we compute the local valid rotamer conformations first, and then combine them as global valid conformations. The suggested algorithm is applied to compute the maximum bottleneck of the deformed tunnels derived from the valid rotamer conformations.

Though the graph-based computation of valid conformation has been researched widely, there have been few attempts to find every valid conformation. By considering the two types of graphs, collision and collision free graphs, with maximal cliques, we efficiently remove the rotamers whose conformations are invalid. The number of possible valid conformations in local area is reduced by removing the conformations having collisions. We compute the global valid conformation by combining local results, so the computation time was feasible regardless of the exponential solution space. We also show the novel method to find the maximum size of the ligand which can pass through the flexible tunnel by using the suggested algorithm.

## Figures and Tables

**Table 1:** Experimental Results on Finding Valid Rotamer Conformations.

PDB Id. (Number of Amino Acids)	Tunnel Id.	Number of Tunnel Adjacent Amino Acids	Number of Conformations in Rotamer Cliques	Number of Valid Rotamer Conformations	Computation Time of Valid Rotamer Conformations (s)
1A52 (479)	72	33	829,440	17,280	0.344
	241	22	15,163,200	20,160	0.266
1B44 (531)	107	17	13,500	2160	0.046
	148	30	11,796,480,000	76,800	1.062
1DDZ (962)	78	24	129,600	17,280	0.234
	109	27	844,800,000	300	0.062
	140	22	691,200	10	0.032
	334	26	936,448,128	3,144,960	40.875
1EA1 (447)	152	24	10,800	124	0.203
1MQF (475)	76	18	139,968	288	0.032
1MXT (499)	97	19	432	32	0.031
	108	13	113,400	3825	0.047
	113	26	8,006,169,600	9504	0.188
2OAR (625)	206	28	14,738,630,400	480	0.078
	215	19	539,320,320	327,680	2.984
	298	18	887,040	295,680	2.656
3DOB (293)	39	13	285,768	37,044	0.297
	43	11	20,412	2646	0.031
	53	11	3888	648	0.015
3RLR (440)	51	8	5520	816	0.016
	142	26	10,036,224	180	0.078
	179	22	2688	56	0.031

**Table 2:** Experimental Results on Finding the Maximum Bottleneck.

PDB Id.	Tunnel Id.	Number of Valid Rotamer Conformations	Tunnel Computation Time for All of Valid Conformations (s)	Maximum Bottleneck (Å)	Maximum Bottleneck Computation Time (s)
1A52	72	17,280	17,343	1.256	0.015
	241	20,160	19,329	1.622	0.031
1B44	107	2160	2270	1.139	0
	148	76,800	76,912	2.111	0.078
1DDZ	78	17,280	21,287	1.098	0.016
	109	300	359	0.926	0
	140	10	12	0.916	0
1EA1	152	124	120	1.006	0
1MQF	76	288	287	1.176	0
1MXT	97	32	32	1.148	0
	108	3825	3256	1.721	0
	113	9504	7842	1.2	0.016
2OAR	206	480	553	1.37	0.015
3DOB	39	37,044	31,183	1.187	0.046
	43	2646	2217	1.731	0
	53	648	607	1.974	0
3RLR	51	816	855	1.321	0.015
	142	180	163	1.041	0
	179	56	49	0.984	0
